# The Finances of a Welsh Hospital

**Published:** 1910-01-29

**Authors:** Leonard D. Rea

**Affiliations:** Secretary and General Superintendent. The Infirmary, Cardiff, Secretary's Office


*To the Editor of* The Hospital.

### THE FINANCES OF A WELSH HOSPITAL.

*To the Editor of* The Hospital.

Dear Sir,—I thank you for your kind report and comment in this week's Hospital on our recent Board of Management meeting, when the question of the overdraft was discussed. Perhaps some explanation of the policy of the Board of Management in this matter would be admissible. The increasing demands of this district, the growth of which (mainly owing to the mining industry) has been phenomenal, have thrust upon the Board the responsibility of not only utilising every bed in the Hospital, but also of embarking on extensions, embracing accommodation for 82 additional beds, with provision for 32 more nurses and 25 more servants, as well as modern hospital adjuncts, including a fine pathological block. These extensions have not only been wholly subscribed for, but have also been instrumental in securing from the Treasury an annual grant of £1,500 towards the formation of a medical school.

It has been found impossible to reduce, even temporarily, the existing number of beds (196), as there are at no time fewer than 600 patients waiting for admission, and, apart from humanitarian reasons, the failure to meet to our utmost the obligation which we owe to the subscribers would have resulted in a diminution of their sympathy and support.

The Board has therefore in the first instance made full provision for increased accommodation, and is now working most strenuously and most successfully with the single object of increasing the income. To give an idea of the success of our appeal for this purpose, I am glad to be able to say that, within the past twelve months, £40,000 has been added to our capital, which now exceeds £100,000, whilst, in addition to the interest which we are enjoying from this sum, the subscription list has been improved by nearly £1,500 a year. The Board is strongly of opinion that an appeal to reduce the overdraft at this juncture would prejudice the success of the appeal for an increased income, whilst it is confidently hoped that the income will be so increased that the overdraft will be diminished without diverting public sympathy from what we firmly believe will prove the radical cure of our financial difficulty.

I may add that this policy has been adopted as a result of local circumstances and experience, and is well understood and approved of by the wealthy friends of the Hospital.

I am, Sir, your obedient servant,
LEONARD D. REA,
Secretary and General Superintendent.
The Infirmary, Cardiff, Secretary's Office,
January 22, 1910.

